# On Phagocytosis

**Published:** 1907-11-16

**Authors:** 


### Points in Pathology.

### ON PHAGOCYTOSIS.

Phagocytosis will always be associated with the name of Metchnikoff. For he it was who, some thirty years ago, demonstrated the phagocytic powers of certain leucocytes and tissue-cells, and claimed that these cells represent the sole protective mechanism of the body against invading organisms. At that time little or nothing was known of the nature of immunity or susceptibility to disease, and Metchnikoff's work was at once so brilliant and so thorough that the evidence in favour of his claim seemed overwhelming, and it was for the time being accepted almost universally as the truth. These discoveries naturally attracted much attention, and induced many other workers to enter so promising a field of research. As a result a number of facts came to light which seemed at variance with Metchnikoff's views, and the idea began to gain ground that the fluid constituents of the body, and of the blood in particular, played a part equal to, or even greater than that of the cells in the destruction of bacteria. It was found that the blood-serum of animals, especially of immunised animals, is possessed of marked bactericidal powers, and at length it came about that the purely "cellular" hypothesis was discarded by many for the purely "humoral" hypothesis, which explained phagocytosis by supposing that any bacteria taken up by cells had previously been killed by the serum. As so often happens, these two extremes of opinion were succeeded by a compromise in the form of the "cellulo-humoral" hypothesis, now commonly accepted, which attributes both to the fluid and cellular elements of the blood and tissues a share in the defensive apparatus of the body.

With the progress of time Metchnikoff, in fact rather than in name, has considerably modified his views, so that they now fall nearly into line with this "cellulo-humoral" conception. For instance, though unable to deny that the blood-serum may have a definite bactericidal action, he would explain this as due to the liberation of a ferment, "cytase," set free in the serum by the "phagolysis," or breaking-up of the leucocytes, which takes place when the blood clots outside the body. Again, "Pfeiffer's phenomenon," consisting of the agglutination and destruction of cholera vibrios introduced into the peritoneal cavity of a guinea-pig immunised against cholera, a destruction occurring without the apparent intervention of any cellular elements, Metchnikoff regards as caused by a similar "cytase" liberated by "phagolysis" of leucocytes collected on the omentum; whereas Pfeiffer thought the vibrios were killed by bactericidal substances secreted by the peritoneal endothelium. These two instances serve to show that, although Metchnikoff still insists on the importance of the cells as originators of bactericidal substances, he no longer regards the action of these substances as limited to the confines of the cells.

It was with the object of determining the relative importance of the corpuscles and the serum of the blood as bactericidal agents that Wright and Douglas undertook their classical experiments which led to the discovery of opsonins, and eventually to the opsonic treatment of various diseases. In these experiments there were three interagents. First, blood-serum freed from leucocytes by centrifugalisation; second, leucocytes freed from serum by centrifugalisation and repeated washing in normal saline solution; third, a suspension of cocci or certain other organisms in normal saline solution. They found that after incubating a mixture of leucocytes, serum, and bacteria at 37° C. for fifteen minutes marked phagocytosis occurs. If, however, the serum is omitted from the mixture, or is heated to 60° C. for ten minutes before mixing, scarcely any phagocytosis takes place. They concluded, therefore, that serum contains certain elements which influence either the leucocytes or the bacteria in such a manner as to enable phagocytosis to occur. By further experiments they proved that these elements are destroyed by heating the serum to 60° C. for ten minutes, and that their action is on the bacteria rather than on the leucocytes. Consequently they termed these elements "opsonins" (from a Greek word meaning to prepare a meal for), because they seem to make the bacteria more palatable to the leucocytes.

These experiments would appear to prove that the leucocytes are of themselves an inert factor in the phenomena of phagocytosis, which seems to depend primarily on the action of the opsonins contained in the serum. However, there has been issued from Metchnikoff's laboratory a paper dealing critically with many points of this work. The authors bring forward evidence to prove that the phagocytic power of the blood varies widely towards different strains of the same organism, that certain organisms are ingested as readily in the absence as in the presence of opsonins, whilst others resist phagocytosis under any conditions. They believe, therefore, that only occasionally does phagocytosis depend on the presence of opsonins, which are then formed by the leucocytes; and that in the great majority of cases the cellular elements still remain the prime factor in repelling bacterial invasion.

It is evident, then, that the last word on phagocytosis still remains to be said, and that the exact significance of the process is far from clear. We can only say that the degree of phagocytosis shown towards any particular organism affords, within certain limits, a valuable index of the power of the body to resist the attacks of that organism, but we are as yet quite unable to apportion to the fluid or cellular elements of the body their exact share in the production of these powers of resistance. The questions of phagocytosis, susceptibility, and immunity, which so closely depend one on the other, are extremely difficult and obscure. They require the solution of very complex bio-chemical problems. Nevertheless this line of research has led to such brilliant results in the past that we may confidently expect them to be continued in the future.

